# The relationship between ambivalence towards supervisor's behavior and employee’s mental health

**DOI:** 10.1038/s41598-022-13533-2

**Published:** 2022-06-10

**Authors:** Raphael M. Herr, Wendy C. Birmingham, Frenk van Harreveld, Annelies E. M. van Vianen, Joachim E. Fischer, Jos A. Bosch

**Affiliations:** 1 Mannheim Institute of Public Health, Social and Preventive Medicine, Medical Faculty Mannheim, Heidelberg University, Mannheim, Germany; 2 Department of Psychology, Brigham Young University, Provo, USA; 3 Faculty of Social and Behavioral Sciences, Social Psychology Program, University of Amsterdam, Amsterdam, The Netherlands; 4 Department of Work and Organizational Psychology, University of Amsterdam, Amsterdam, The Netherlands; 5 Department of Clinical Psychology, University of Amsterdam, Amsterdam, The Netherlands

**Keywords:** Psychology, Risk factors, Psychiatric disorders

## Abstract

Ambivalence in social interactions has been linked to health-related outcomes in private relationships and recent research has started to expand this evidence to ambivalent leadership at the workplace by showing that ambivalent supervisor-employee relationships are related to higher stress levels in employees. However, the mental health consequences of ambivalent leadership have not been examined yet. Using a multilevel approach, this study estimated associations of ambivalent leadership with mental health indicators (depression, anxiety, vital exhaustion, fatigue) in 993 employees from 27 work groups. A total effect of ambivalent leadership was found for all four mental health measures, as well as within-group and between-group effects. The consistent relationships of ambivalent leadership with higher symptoms of mental ill-health at the individual- (i.e., within-group) and the group-level (i.e., between-group) support the existence of an un-confounded association, as well as group effects of collective ambivalence.

## Introduction

Individuals often simultaneously carry positive and negative attitudes, feelings, and beliefs towards an object or person, which is denoted as ambivalence^1^. Ambivalence is inherent to most—if not to a certain extent to all—social relationships and may have negative effects. The ABC (Affect, Behavior, Cognition) model of ambivalence, for example, predicts that people are motivated to be internally consistent whereby ambivalence is experienced as unpleasant, leading to distress and negative affect^2^. Likewise, the Stress Enhancing Hypothesis predicts that ambivalence in relationships will cause emotional distress^3^, and the Social Ambivalence and Disease (SAD) model additionally proposes that ambivalent social relationships are not helping much in coping with stress and provide little social support^4^. The above theoretical predictions are confirmed by a growing body of evidence, based on both experimental and observational studies, showing that ambivalent social ties in personal relationships (e.g., partners, friends, and family) are negatively linked to psychological and physical health-related outcomes. These include greater psychological distress and reduced mental health, as well as higher blood pressure, cardiovascular responses, shorter telomere length, and higher levels of inflammatory markers^2,5–14^. Combining the theoretical considerations and empirical findings, it seems justified to assume that ambivalence in social relationships leads to negative health outcomes through persistently elevated distress levels.

While ambivalence is commonly considered pernicious, studies of emotional ambivalence (i.e., mixed emotions in terms of co-occurrences of positive and negative emotions) have reported positive associations between ambivalence and well-being and physical health^15–17^. These positive effects can be explained by Larsen and colleagues’ co-activation model^18^, which states that, people adopt a strategy ‘‘to take the good with the bad’’. By finding meaning in negative events, they are better able to cope with adversity, which can positively affect their health.

To date, ambivalent social ties in personal relationships have been relatively less studied in the context of work, while ambivalence and ambivalent supervisor-employee relationships in particular are very likely to occur in the workplace^19,20,22,23^, which may affect employee well-being. Such an ambivalent supervisor-employee relationship may manifest itself, for example, as ambivalent leadership when subordinates consider the behavior of their leader to be both supportive and burdensome at the same time^21^. Based on existing empirical evidence of the detrimental health effects of ambivalent personal relationships, it is plausible to assume negative health effects of such ambivalent supervisor-employee relationships in the workplace. In a recent study we found that supervisor behavior perceived by subordinates as ambivalent was associated with higher levels of subordinates’ self-reported stress and the stress hormone cortisol^21^. Moreover, a study by Ciampa and colleagues^22^ provides an indication of negative health effects by showing that employees’ ambivalent identification with the organization was associated with higher levels of exhaustion.

Regarding motivational and behavioral outcomes, ambivalent leadership may have positive consequences as well^19^. For example, ambivalent leadership can promote adaptive or proactive behavior, engagement, and performance^23^. These positive consequences seem to result primarily from simultaneous positive and negative emotions expressed by the leader about complex projects or strategic initiatives^24–27^. However, ambivalence expressed by the leader could also reduce employees’ engagement and performance^28,29^. Based on emotions as social information (EASI) theory^30^, Lim and colleagues^29^ argued and showed that supervisor’s expressed emotional ambivalence induces employees’ perceptions of supervisor’s unpredictability and anticipated stress, which in turn, reduce employees’ task engagement, with the strongest reduction when the supervisor’s ambivalence was directed at themselves and not at another subordinate. Guarana, Rothman and Melwani^31^ describe leader ambivalence as “a double-edged” sword that produces both detrimental and beneficial effects on subordinate’s task performance. They suggest that the effects depend on project complexity and the extent to which leaders and their team show information seeking behaviors.

While ambivalence at the workplace might have positive as well as negative effects on engagement and task performance, in the current study, we focus on the potential negative effects of leadership behavior perceived as ambivalent by subordinates on subordinates’ mental health. Specifically, we are interested in whether the mental health of subordinates who share the same working environment is threatened if they perceive that their leader behaves both positively and negatively at the same time. We examine the relationship between employees’ ambivalent leadership and employees’ mental health, such as depressed mood, anxiety, fatigue, and vital exhaustion, which are known consequences of prolonged high stress levels.

Ambivalence may be experienced at both the individual and the group level. Group (or collective) ambivalence is defined as a simultaneously positive and negative orientation (e.g., emotions, cognitions) toward an object or person (e.g., a supervisor) experienced by a collective of persons^32,33^. According to Pradies and Pratt^32^, group ambivalence resides in the interactions of its members, by which individual emotions and thoughts become group-level emotions and thoughts. Collective ambivalence can thus be seen as a phenomenon of distributed cognitions and a common property of social networks^33^. It is conceivable, then, that collective ambivalence may act as a contextual factor that has additional effects on individual health outcomes. We have previously demonstrated such contextual effects for organizational justice climate and mentoring climate^34,35^, i.e., showing that group level perceptions explain additional variance in individual outcomes above and beyond individual level perceptions. The existence of such contextual effects could imply that interventional strategies may be more efficacious when they target both the individual and the collective level.

Simultaneously studying the individual and collective level has the additional value that it may help identify significant reporting bias. Especially cross-sectional assessments and studies utilizing questionnaire data for both the exposure and dependent variables are at risk of yielding spurious results^36^. It is, for example, possible that employees’ health can confound their perception of their supervisor’s behavior, or that individual-level relationships are biased by contextual effects. The impact of such confounding can be identified by analyzing data at both the individual- and the group-level whereby a significant effect at both levels of analysis is indicative of a reduced risk of bias^37,38^.

The aim of the present study is to investigate the association between ambivalent leadership and indicators of mental health (i.e., symptoms of depression, anxiety, fatigue, and exhaustion). These associations are analyzed at both the individual (within-group) and collective level (between-group) to estimate individual and contextual effects.

## Results

The 993 participants worked in 27 work groups with a median size of 29 persons (interquartile range = 47.5). Most participants were male (88.3%) with an average age of 42 years (standard deviation = 10.6). The majority had a job description categorized as blue-collar occupation (67.7%) and 36.6% engaged in shift work (Table 1).

**Table 1:** Sample characteristics.

	Mean or %	n or SD
**Demographic factors**		
Male	88.3%	877
Age (years)	41.9	10.6
**Job characteristics**		
Job position		
Division/department manager	17.6%	175
Project, group leader/process manager	75.7%	752
Skilled/semi-skilled worker	6.7%	66
Work schedule		
No shift work	63.4%	630
Shift work	36.6%	363
Type of occupation		
Blue-collar	67.7%	672
White-collar	32.3%	321
**Lifestyle factors**		
Smoking		
Never smoker	40.6%	403
Ex-smoker	29.3%	291
Smoker	30.1%	299
Alcohol consumption (mean gr/day)	19.0	26.2
Physical activity		
Regularly > 2 h/week	27.4%	272
Regularly 1–2 h/week	28.5%	283
Regularly < 1 h/week	17.5%	174
No physical activity	26.6%	264
Body Mass Index (BMI; mean)	24.4	3.8
**Mental health**		
Depression (0: low–21: high)	4.7	3.5
Anxiety (0: low–21: high)	6.0	3.5
Fatigue (1: low–5: high)	2.7	0.7
Exhaustion (1: low–5: high)	2.5	0.7

Correlational analysis showed negative associations between positive leadership behavior and indicators of low mental health (*r* between − 0.19 and − 0.28), and a positive association between negative leadership behavior and these indicators (*r* between 0.24 and 0.30; Table 2).

**Table 2:** Correlation between key variables.

	Ambivalence	Positivity	Negativity	Depression	Anxiety	Fatigue
Positivity	− 0.24					
Negativity	0.49	− 0.59				
Depression	0.16	− 0.28	0.26			
Anxiety	0.18	− 0.19	0.25	0.69		
Fatigue	0.16	− 0.23	0.24	0.60	0.57	
Exhaustion	0.18	− 0.26	0.30	0.68	0.66	0.76

Ambivalent leadership was operationalized by combining employee ratings of positive and negative supervisor behaviors into an index applying the formula developed by Griffin^39^, in which higher values indicate more ambivalence. As shown in Table 3, an overall effect of ambivalent leadership was found for each of the four mental health measures (total effect betas, adjusted for sex, age, job characteristics, and lifestyle factors: ≥ 0.17, all p-values < 0.001). When further partitioned, a within-group effect (betas: ≥ 0.16, all p-values < 0.001), and between-group effect was found for each of the mental health measures (betas ≥ 0.34, p-values ≤ 0.009). Test of heterogeneity revealed significant difference between the within- and between-group effects (p-values < 0.001), specifying a contextual effect of collective ambivalence. Effects became somewhat attenuated when additionally controlling for the individual positive and negative behavior scales, but generally remained significant (Model 3). Regarding the control variables, the following variables had a significant effect (p < 0.005) in the full adjusted models (Model 3). For depression: job position, alcohol consumption, and physical activity; for anxiety: alcohol consumption; for fatigue: age, work schedule, physical activity, and BMI; for exhaustion: age, alcohol consumption, and physical activity.

**Table 3:** Association of ambivalence with mental health indicators.

Outcome	Ambivalent leadership								
	Model 1			Model 2			Model 3		
	Beta	S.E	p-value	Beta	S.E	p-value	Beta	S.E	p-value
**Depressive symptoms**									
Total effect	0.17	0.04	< 0.001	0.17	0.04	< 0.001	0.08	0.03	0.003
Within-group effect	0.15	0.04	< 0.001	0.16	0.04	< 0.001	0.07	0.03	0.007
Between-group effect	0.35	0.10	< 0.001	0.34	0.07	< 0.001	0.17	0.07	0.01
Test of heterogeneity			< 0.001			< 0.001			0.01
**Anxiety**									
Total effect	0.17	0.04	< 0.001	0.18	0.04	< 0.001	0.09	0.04	0.02
Within-group effect	0.16	0.04	< 0.001	0.16	0.04	< 0.001	0.09	0.04	0.03
Between-group effect	0.48	0.10	< 0.001	0.50	0.10	< 0.001	0.34	0.09	< 0.001
Test of heterogeneity			< 0.001			< 0.001			< 0.001
**Fatigue**									
Total effect	0.16	0.03	< 0.001	0.18	0.03	< 0.001	0.09	0.03	0.003
Within-group effect	0.15	0.03	< 0.001	0.17	0.03	< 0.001	0.09	0.03	0.005
Between-group effect	0.32	0.15	0.03	0.36	0.14	0.01	0.19	0.13	0.13
Test of heterogeneity			0.03			0.01			0.13
**Exhaustion**									
Total effect	0.19	0.04	< 0.001	0.19	0.04	< 0.001	0.08	0.03	0.01
Within-group effect	0.17	0.04	< 0.001	0.17	0.04	< 0.001	0.07	0.03	0.02
Between-group effect	0.46	0.11	< 0.001	0.41	0.12	< 0.001	0.20	0.11	0.07
Test of heterogeneity			< 0.001			< 0.001			0.07

Model 1 = adjusted for sex and age.

Model 2 = adjusted for sex and age, and job characteristics and lifestyle factors.

Model 3 = adjusted for sex and age, and job characteristics and lifestyle factors, and positive (positivity) and negative (negativity) supervisor behavior.

Test of heterogeneity: Hausman test for difference between within-group effect and between-group effect.

## Discussion

The adverse effects of a negative leadership style on health, as well as the positive effects of a positive and supportive style are well established e.g.,^40–42^. Consistent with theoretical predictions^2,4^, the present study was the first to identify that the combination of these two—denoting ambivalent leadership—represents a risk factor for poor mental health at the workplace. Ambivalence was associated with higher depressed mood, anxiety, fatigue, and vital exhaustion. These associations were found both at the individual (i.e., within-group) and the group level (i.e., between-group). Further, an additional contextual effect indicated that group-level perceptions of ambivalent leadership predicted health beyond the individual-level effect. This contextual effect denotes that employees who have the same perception of ambivalent leadership but belong to work groups that vary in collective (i.e., group mean) ambivalent leadership differ in mental health. This means, for example, that employees’ health is negatively affected when their team members experience more ambivalent leadership than they do. One possible implication of this finding is that potential workplace interventions aiming to prevent mental health by reducing leadership ambivalence may become more effective when group-level processes are considered. Existence of both a within- and between-effect is indicative of an unbiased association, providing confidence in the robustness of the findings. These novel observations extend recent findings by our prior research showing that ambivalent leadership perceptions were associated with higher self-reported stress and higher levels of the stress hormone cortisol^21^.

Notably, employees’ ratings of supervisor’s positive and negative behaviors and ambivalent leadership were independently related to health outcomes, indicating that their combination is most predictive for employees’ health. This observation may likewise have implications for intervention designs, as it suggests that the effects of negative leadership behaviors on employee health cannot be simply compensated by positive leadership behaviors, as it is their combination that yield an additional effect.

A key aspect that may play a role in the extent to which mental health might be impaired is the degree of ambivalence^19^. In this study, ambivalent leadership was defined as the simultaneous perception of supportive and burdening leader behaviors. This ambivalent leadership refers to behavior of closely related dimensions, which have the potential to raise high degrees of experienced ambivalence in people^19^. However, further studies are needed to test the effects of different behavioral dimensions in ambivalent supervisor-employee relationships.

Reaching the boundaries of this study, there are several aspects that should be considered in further research. First, whether supervisor-employee ambivalence has positive or negative consequences may depend on moderating factors like gender, self-esteem, organizational support, and quality of work-life^19^. Moreover, based on the conservation of resources (COR) theory^43^, Zhao and Zhou^19^ argue that ambivalence as “threat of resources loss” will lead to uncertainty, unpredictability, and negative outcomes such as negative affect, strain, and ill-health, while ambivalence as “opportunity to gain resources” will result in positive outcomes, like flexibility and engagement. Specifically, they propose three moderators that influence employees’ positive or negative outcomes of ambivalence: degree of ambivalence, support from a third party, and integrative complexity of the employee. The higher the degree of ambivalence (e.g., supervisor behavior is viewed as both undermining and supportive) the more likely it is that employees will experience loss of resources, resulting in negative employee outcomes. In this study, leadership ambivalence was defined by the combination of both positive and negative leadership behaviors, that is, supportive and burdensome leader behaviors as experienced by subordinates. This ambivalence is associated with negative outcomes, like lower mental health. Furthermore, employees who experience (third party) social support are more likely to get positive outcomes because this support will alleviate the stress resulting from supervisor-employee ambivalence and it will help employees to interpret the ambivalence in a more integrative way. In this study, we did not measure social support from others than the supervisor, and the moderating influence of third-party support could therefore not be considered here. Leaders and peers (members of the work team) are important sources of employees’ experienced social support. In this study, we found a contextual effect of leadership ambivalence, meaning that employees who share their perceptions of high leadership ambivalence with their peers may suffer rather than benefit from peer support, which may be due to emotional contagion (i.e., the transfer of emotions among people in a group)^44^. Lastly, employees with higher integrative complexity, that is, with a greater capacity and willingness to acknowledge conflicting perspectives, are more likely to experience positive outcomes. Since an individual tendency to allow for conflicting perspectives was not considered in this study, further studies are needed to examine this possible moderator.

Next to behavior-based ambivalence—studied here—many other forms are conceivable and exist and might raise different effects. A recent review identified ten different types of ambivalent supervisor-employee relationships by classifying their source (affect-based, cognition-based, multiplex/complex ties, and behavior) and their cross-ambivalence^19^. Future studies could systematically investigate the positive and negative effects of these types, taking into account various moderators.

Other interesting further research directions are presented by Guarana and Hernandez^27^ and Melwani and Rothman^45^. Guarana and Hernandez^27^ examine shared ambivalence of leader and subordinate. The authors develop a theoretical model for ambivalence becoming a functional cognitive process with a joint interpretation when leaders and employees share ambivalent cognitive states. Melwani and Rothman^45^ focus on emotional ambivalence regarding co-workers (frenemies), probing when persons react with helpful and when with harmful behaviors. Thus, future research could investigate whether emotional ambivalence regarding co-workers could explain additional variance in employee health outcomes beyond ambivalent leadership.

This study has several limitations. First, the cross-sectional design of this study does not allow causal relationships to be inferred. Second, the scales measuring positive and negative leadership behaviors were restricted. That is, negative leadership behavior was conceptualized as burdening and positive leadership behavior as supportive, while leadership behavior comprises a wider range and variety of characteristics that were not assessed in this study.

In conclusion, this study is the first to provide evidence for an association of individual and collective ambivalent leadership with indicators of lower mental health. Ambivalent leadership thus represents a hitherto neglected risk factor for mental health at the workplace, and further research is warranted to identify its toxic components and establish the generalizability of our findings.

## Methods

### Study population

The present cross-sectional study involved employees of a large manufacturing company in the South of Germany who participated in a health check in 2007. Volunteer participants completed questionnaires covering e.g., demographic, health behavior, work status, work characteristics, mental health, and underwent physical examinations. Individual work group affiliation was obtained from personnel services. Complete data were available for 993 employees, involving 27 work groups. All participants provided written informed consent prior to participation, the ethics committee of the Medical Faculty Mannheim of the University Heidelberg approved the study (2007-009E-MA), and the study was carried out in accordance with the Code of Ethics of the World Medical Association (Declaration of Helsinki).

### Ambivalent leadership

**Figure 1:**
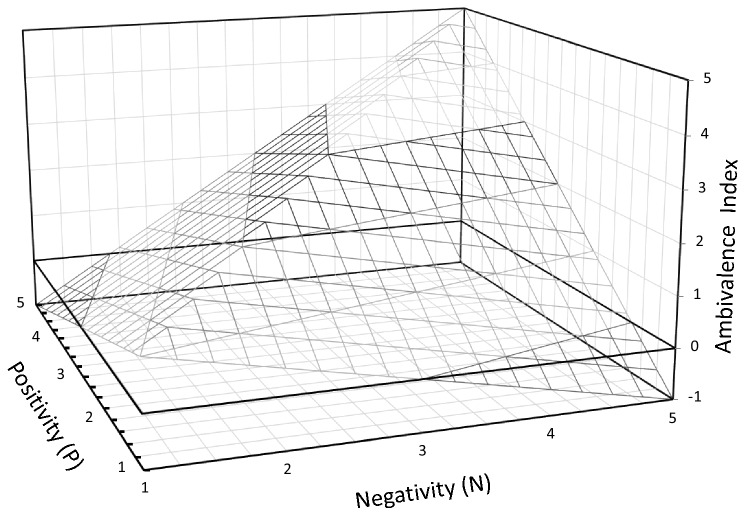
Schematic representation of the ambivalence index.

In line with previous research and as standard practice in ambivalence research e.g.^2,21^, ambivalent leadership was determined by combining positive and negative ratings of supervisor behavior into a single ambivalence index applying the formula developed by Griffin^39^: (P + N)/2 − |P − N|, whereby P = positivity (i.e., mean of positive behavior) and N = negativity (i.e., mean of negative behavior). This formula takes into account the theoretical assumption that ambivalence rises with the similarity in magnitude and the intensity of the positive and negative components^39^. In consequence, the ambivalence index is highest if the positive and negative component are high in intensity (i.e., at the high end of the scale) and similar in their content (i.e., positive and negative scale values are as close together as possible). The result is a continuous index with higher values representing higher leadership ambivalence and lower values lower leadership ambivalence (Fig. 1). Positive leadership behavior was measured by five items (Cronbach’s α = 0.81) and negative behavior by three items (Cronbach’s α = 0.78) from the Salutogenetic Subjective Work Analysis questionnaire^46^. All items were rated on a 5-point Likert scale ranging from 1 (does not apply at all) to 5 (fully applies). For both scales mean scores were computed. Example items for positive leadership behavior were “The direct supervisor lets you know how well you did your work”. Negative behavior was assessed by items like “If a mistake is made, the supervisor puts all the blame on us, never on himself/herself”. Collective ambivalent leadership was defined as the mean of individual ambivalent leadership within a work group (intraclass correlation coefficient [i.e., proportion of the total variance explained by the grouping structure] = 9.7%; mean within-group interrater agreement index [rwg(j)]^47^ = 0.772).

### Mental health indicators

Depression and anxiety symptoms were measured by the corresponding subscales of the German version of the Hospital Anxiety and Depression Scale (HADS)^48^. Both the depression and the anxiety subscale consist of seven items, to be answered on a 4-point Likert scale ranging from 0 = mostly to 3 = not at all (Cronbach’s α anxiety = 0.80; depression = 0.81). Sum scores were computed for each scale, ranging from 0 (low) to 21 (high).

Vital exhaustion was assessed with the Shortened Maastricht Exhaustion Questionnaire (9 items; Cronbach’s α = 0.90)^49,50^ and fatigue by 9 items (Cronbach’s α = 0.85) of the Need for Recovery Scale^51^. All items were rated on a 5-point Likert scale from 1 = always to 5 = never. A reversed mean score was computed implying that higher values indicate more fatigue or exhaustion, respectively.

### Statistical analysis

**Figure 2:**
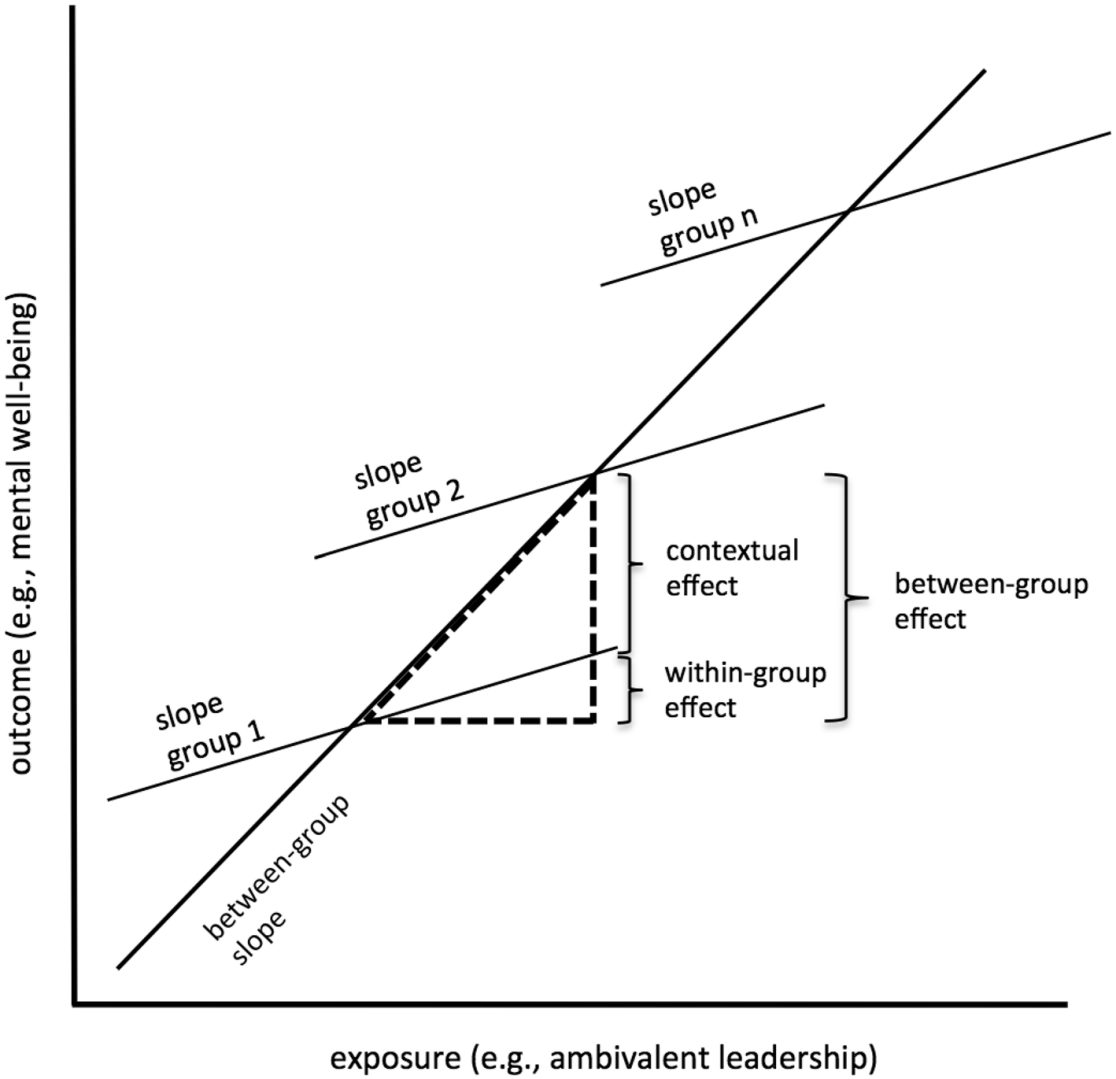
Conceptual description of *within-group effects* (i.e., mental health among employees that belong to the same work group as predicted by their ambivalent leadership perceptions), *between-group effects* (i.e., mean levels of mental health of work groups as predicted by the group-mean ambivalent leadership perceptions), and *contextual effects* (i.e., mental health of employees that have the same ambivalent leadership rating, but belong to work groups that differ in their mean ambivalent leadership ratings). This contextual effect is demonstrated by significant heterogeneity (Hausman test).

Multilevel linear regression models were applied to estimate the associations of ambivalent leadership with, respectively, depression, anxiety, fatigue, and exhaustion. In a first step, a model only including the individual level exposure estimated the total effect of ambivalent leadership on the outcomes, considering the membership to a work group as a random slope effect. In the next step, this total effect was disassembled into a between-group (i.e., the mean of the group) and a within-group (i.e., the individual deviation from the group mean) effect (cf. Fig. 2). To test whether disassembling the total effect is appropriate, an alternative parameterization with the individual exposure instead of deviation from the group mean was used. A significant test of heterogeneity (Hausman test) specifies whether both effects are different and indicates the presence of a contextual effect beyond the individual level association (i.e., the difference between the between-group and the within-group effect, cf. Fig. 2)^37^. Variables were Z-transformed to obtain standardized regression coefficients (i.e., betas) and covariates were included as fixed effects. Three models of gradual adjustment were estimated. The first models were adjusted for age and sex. The second set of models was additionally adjusted for lifestyle factors (i.e., smoking, alcohol consumption, physical activity, and BMI), and job characteristics (i.e., job position, work schedule, and type of education), while the third set also controlled for the separate positive and negative supervisor behavior scales. All statistical analyses were performed using StataSE 14 (StataCorp, College Station, TX).

## Data Availability

Due to the legal data protection regulations, the data cannot be made publicly accessible. The code can be requested from the corresponding author.
